# Predictive Modeling of Colonoscopic Findings in a Fecal Immunochemical Test-Based Colorectal Cancer Screening Program

**DOI:** 10.1007/s10620-021-07160-6

**Published:** 2021-08-04

**Authors:** Jade Law, Anand Rajan, Harry Trieu, John Azizian, Rani Berry, Simon W. Beaven, James H. Tabibian

**Affiliations:** 1 UCLA-Olive View Internal Medicine Residency Program, Sylmar, CA USA; 2 Keck School of Medicine, University of Southern California, Los Angeles, CA USA; 3 UCLA Internal Medicine Residency Program, Los Angeles, CA USA; 4 Vatche and Tamar Manoukian Division of Digestive Diseases, David Geffen School of Medicine at UCLA, Los Angeles, CA USA; 5 Division of Gastroenterology, Olive View-UCLA Medical Center, Sylmar, CA USA

**Keywords:** Early detection of cancer, Colonoscopy, Adenoma, Colorectal neoplasms, Machine learning, Social class

## Abstract

**Background:**

The fecal immunochemical test (FIT) is the primary modality used by the Los Angeles County Department of Health Services (LADHS) for colorectal cancer (CRC) screening in average-risk patients. Some patients referred for FIT-positive diagnostic colonoscopy have neither adenomas nor more advanced pathology. We aimed to identify predictors of false-positive FIT (FP-FIT) results in our largely disenfranchised, low socioeconomic status population.

**Methods:**

We conducted a retrospective study of 596 patients who underwent diagnostic colonoscopy following a positive screening FIT. Colonoscopies showing adenomas (or more advanced pathology) were considered positive. We employed multiple logistic and linear regression as well as machine learning models (MLMs) to identify clinical predictors of FP-FIT (primary outcome) and the presence of advanced adenomas (secondary outcome).

**Results:**

Overall, 268 patients (45.0%) had a FP-FIT. Female sex and hemorrhoids (odds ratios [ORs] 1.59 and 1.89, respectively) were associated with increased odds of FP-FIT and fewer advanced adenomas (*β* = − 0.658 and  − 0.516, respectively). Conversely, increasing age and BMI (ORs 0.94 and 0.96, respectively) were associated with decreased odds of FP-FIT and a greater number of advanced adenomas (*β* = 0.073 and 0.041, respectively). MLMs predicted FP-FIT with high specificity (93.8%) and presence of advanced adenoma with high sensitivity (94.4%).

**Conclusion:**

Increasing age and BMI are associated with lower odds of FP-FIT and greater number of advanced adenomas, while female sex and hemorrhoids are associated with higher odds of FP-FIT and fewer advanced adenomas. The presence of the aforementioned predictors may inform the decision to proceed with diagnostic colonoscopy in FIT-positive patients.

## Introduction

Colorectal cancer is the second leading cause of cancer-related death in the United States and accounts for 10% of all cancer-related death worldwide [1]. Early detection through screening programs has proven essential in reducing cancer mortality [2]. While colonoscopy is considered the gold standard for early detection of CRC, the cost and cumbersome nature of procedures have led to more frequent use of noninvasive initial screening tests. The fecal immunochemical test (FIT) is the primary modality used in the Los Angeles County Department of Health Services (LADHS) for asymptomatic, average-risk patients. At a hemoglobin cutoff of 100 ng/mL or 20 ug/g, the specificity of FIT tests for CRC is 95% and for advanced neoplasia 97% [3].

Despite these promising statistics, 39–52% of patients referred for FIT-positive diagnostic colonoscopy have neither adenomas nor more advanced pathology [4]. Such false-positive FIT (FP-FIT) results expose patients to unnecessary colonoscopy, which increases healthcare burden and cost, exposes patients to unnecessary interventions, reduces patient compliance to yearly FIT testing [5], and can generate psychological distress up to 6 weeks after a normal colonoscopy [6]. This directly hampers efforts to reduce unnecessary health care interventions.

Previous studies have examined factors affecting FP-FITs with conflicting findings. Some studies suggest that pharmacologic agents such as aspirin, clopidogrel, warfarin, and nonsteroidal anti-inflammatory drugs (NSAIDs) have no impact on FIT test characteristics [7–10]. Other studies, however, suggest that one or more of these medications significantly impact FIT results [9, 11–13]. There are also disparities as to whether factors such as age, sex, presence of hemorrhoids, smoking history, CRC history, or BMI influence FP-FIT results [4, 11, 14–23].

Given the contradictory evidence from previous studies and lack of individual studies comparing multiple factors within the same population, we aimed to identify demographic, personal, pharmacologic, and other clinical predictors that lead to FP-FIT in our largely Hispanic LADHS population. Identifying these factors can aid in creating personalized screening strategies, strengthening conclusions gained from FIT results, and reducing unnecessary colonoscopies. Finally, we trained multiple machine learning models (MLMs) to predict FP-FIT as well as the presence of advanced adenoma and compared their performance.

## Materials and Methods

### Study Population

We conducted a retrospective study of average-risk patients at or over the age of 50 who underwent diagnostic colonoscopy following a positive screening FIT between 2015 and 2018 at Olive View-UCLA Medical Center (OVMC), one of three major hospitals within LADHS. Average-risk patients were asymptomatic individuals without family history of colorectal cancer or prior premalignant or malignant polyps. A total of 596 adult patients were identified, all of whom had undergone FIT screening with the OC-Auto-FIT test, an immunochemical test using a hemoglobin level of 100 ng/mL (20 ug/g) as the threshold for a positive FIT.

### Endoscopic and Pathologic Procedures and Definitions

All colonoscopies were performed at OVMC. Participants prepared for colonoscopy per standardized instructions, including: a clear liquid diet the day before endoscopy and completing four liters of split-dose polyethylene glycol solution (GoLYTELY) the evening prior to endoscopy. Colonoscopies were excluded if deemed by the performing endoscopist to have suboptimal or inadequate bowel preparation. The study only included colonoscopies demonstrating adequate preparation. All visualized lesions were biopsied or removed and sent for histologic assessment.

Data on endoscopic and histologic findings were collected, including the number of adenomatous polyps and the size of the largest polyp found. Colonoscopies demonstrating one or more adenomas or more advanced pathology were defined as positive. Advanced adenomas were classified according to recent societal guidelines (adenomas with size greater than 10 mm, three or more adenomas, or histology showing tubulovillous or villous morphology or adenocarcinoma) [31]. Colonoscopies demonstrating only hyperplastic polyps were defined as negative (i.e., FP-FIT).

### Predictor Variables

Predictors collected from each patient’s electronic health records and colonoscopy reports included: age, sex, ethnicity, body mass index (BMI), history of smoking, personal history of gastrointestinal malignancy, presence of diverticula on colonoscopy, presence of hemorrhoids, NSAID use, antiplatelet agent use, and anticoagulation use. Medications were only included if actively used by the patient at the time of positive FIT testing as evidenced by clinic visit notes.

### Statistical Analysis and Machine Learning Models

We performed descriptive statistics to depict the patient population and compare characteristics between patients with and without a FP-FIT (primary outcome). We used multiple logistic regression to investigate relationships between the aforementioned predictors and a FP-FIT result. In addition, we used linear regression to elucidate the relationship between the same predictors and the number of adenomatous polyps observed on colonoscopy (secondary outcome). Patients missing data for any of the predictors were excluded from regression modeling.

Next, we trained machine learning models (MLMs) to predict a FP-FIT result as well as the presence of ≥ 1 advanced adenoma (secondary outcome). The goal was to create a statistical model that could inform the clinician of the presence or absence of an FP-FIT result or, conversely, an advanced adenoma using readily available demographic and clinical parameters. MLMs have been used in recent years to predict CRC using noninvasive parameters such as complete blood count and fecal microbiota composition [24–30]. The statistical modeling improves as the number of data points or patients it is “trained” on grows. A subset of the dataset is traditionally held out to measure the performance of the model (testing set). Because the model is not trained on this subset of the dataset, an understanding of how accurately the model will predict a particular outcome in new, previously unseen patients can be gained.

Our dataset was randomly divided with 80% of observations assigned to the training set and the remaining 20% to the testing set. The FP-FIT training set consisted of 470 patients, of which 212 (45.1%) had a FP-FIT result and 258 (54.9%) had a true-positive FIT (TP-FIT) result. The testing set included 117 patients, of which 64 (54.7%) had a FP-FIT and 53 (45.3%) had a TP-FIT. The advanced adenoma training set consisted of 472 patients, of which 148 (31.4%) had an advanced adenoma and 324 (68.6%) did not, while the testing set was comprised of 117 patients, of which 36 (30.8%) had an advanced adenoma and 81 (69.2%) did not.

We trained the following four supervised MLMs on both the FP-FIT and advanced adenoma data: (1) generalized linear model (GLM), (2) support vector machine (SVM) with linear kernel, (3) SVM with radial basis function (RBF) kernel, and (4) random forest. The same predictors described previously were used as predictors or features in each of the MLMs. Nine patients were excluded from the FP-FIT dataset and seven patients were excluded from the advanced adenoma dataset because data were missing for one or more features. Imputation was not performed as these patients comprised only 1.5% and 1.2% of the entire cohort, respectively. We used tenfold cross-validation for resampling when tuning the SVM and random forest model hyperparameters. Each MLM was then validated on the FP-FIT or advanced adenoma testing sets, and receiver operator characteristic (ROC) curves with corresponding area under the ROC curve (AUROC) were generated.

Youden’s index and the point closest to (0,1) method were used to calculate the optimal cut points above which a patient was considered to have a FP-FIT or advanced adenoma. Youden’s index maximizes the sum of sensitivity and specificity, while the point closest to (0,1) method minimizes the Euclidean distance between the ROC curve and the (0,1) point [32]. Accuracy, sensitivity, specificity, positive predictive value (PPV), and negative predictive value (NPV) were calculated for the best-performing MLM at its optimal cut point.

Descriptive statistics were performed using Stata/IC 16.1 (StataCorp, College Station, TX, USA). Logistic regression, linear regression, and machine learning experiments were performed using R 4.0.2 and the caret, ranger, and pROC libraries. A *p* value < 0.05 was considered statistically significant.

## Results

### Characteristics of the Study Sample

Our study included a total of 596 participants, 268 (45.0%) of whom had a FP-FIT. The main characteristics of the study population stratified by FP and TP results are shown in Table 1. Patients of Hispanic ethnicity constituted greater than half of patients with both FP (58.6%) and TP (58.8%) FIT results. More women (*n* = 169) than men (*n* = 99) had FP results, while a similar proportion of men and women had TP results. Mean age was 59 years in the FP group and 61 in the TP group. The majority of patients in both groups were non-smokers. Less than 4% of both TP and FP patients had a personal history of GI cancer. Fifty-three patients (19.9%) who had FP results were prescribed NSAIDs at the time of FIT, and 80 (30.0%) were on an anti-platelet agent. Hemorrhoids were visualized in 199 (74.3%) and diverticula in 93 (34.7%) patients with FP-FIT during colonoscopy. In patients with a TP-FIT, the median number of adenomatous polyps was two, and the median size of the largest polyp was 1.0 cm.

**Table 1:** Features of consecutive patients who underwent diagnostic colonoscopy following a positive fecal immunochemical test (FIT) result

	False-positive FIT (*n* = 268)	True-positive FIT (*n* = 328)
*Demographics*		
Age, median (IQR)	59.0 (55.0–63.0)	61.0 (57.0–65.0)
Female, *n* (%)	169 (63.1%)	167 (50.9%)
Hispanic, *n* (%)	157 (58.6%)	193 (58.8%)
BMI, median (IQR)	29.6 (26.0–33.0)	30.6 (27.0–35.3)
Never smoker, *n* (%)	190 (71.2%)	210 (64.0%)
History of GI cancer, *n* (%)	10 (3.8%)	10 (3.1%)
History of non-GI cancer	19 (7.1%)	25 (7.7%)
*Medications*		
NSAIDs use, *n* (%)	53 (19.9%)	56 (17.1%)
Anti-coagulant use, *n* (%)	9 (3.4%)	18 (5.5%)
Anti-platelet agent use, *n* (%)	80 (30.0%)	109 (33.2%)
*Endoscopic and histologic findings*		
Diverticula, *n* (%)*	93 (34.7%)	134 (40.9%)
Hemorrhoids, *n* (%)*	199 (74.3%)	199 (60.7%)
Number of polyps, median (IQR)	0.0 (0.0–0.5)	2.0 (1.0–4.0)
Number of adenomatous polyps, median (IQR)	0.0 (0.0–0.0)	2.0 (1.0–3.0)
Size of largest polyp, median (IQR)	0.0 (0.0–0.0)	1.0 (1.0–2.0)

*Presence of internal or external hemorrhoids and diverticula were observed on colonoscopy

### Multiple Logistic Regression Identifies Predictors of FP-FIT Results

**Fig. 1:**
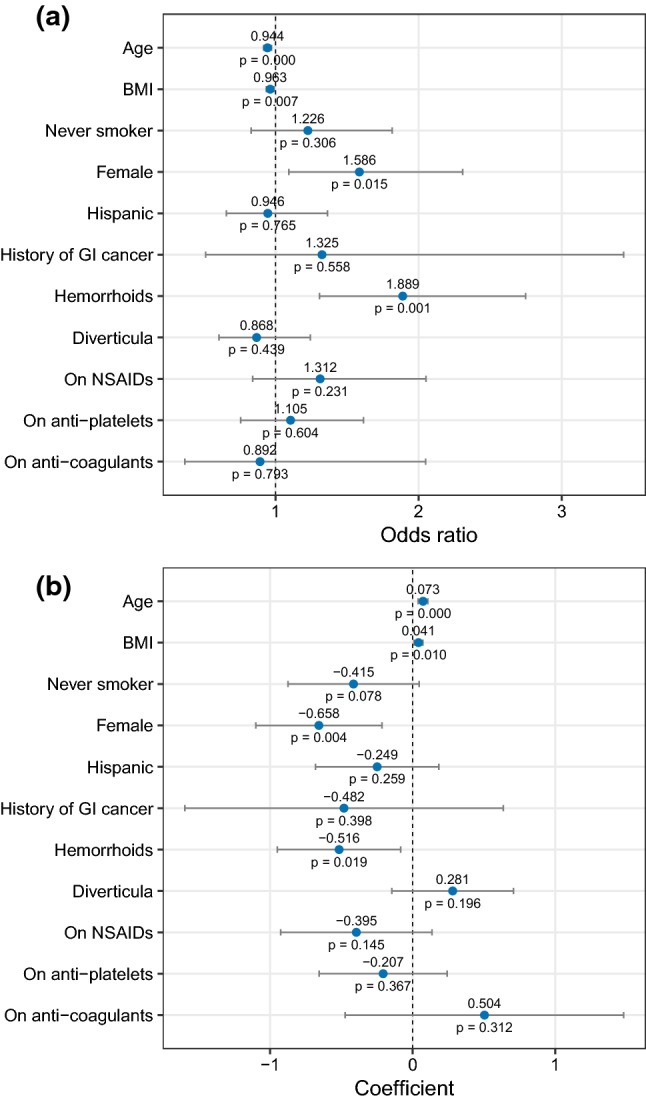
Predictors of an FP-FIT result modeled using logistic regression (**a**) and number of polyps modeled using linear regression (**b**)

As shown in Fig. 1, female gender (OR 1.64, *p* = 0.010) and presence of hemorrhoids (OR 1.91, *p* = 0.001) were associated with increased odds of a FP-FIT. Each year increase in age was associated with a 5.6% decrease in odds of a FP-FIT result (OR 0.94, *p* = 0.000). Similarly, each one unit increase in BMI was associated with a 4% decrease in odds of a FP-FIT (OR 0.96, *p* = 0.007). History of smoking, Hispanic ethnicity, personal history of GI cancer, use of NSAIDs, use of anti-platelet agents, use of anti-coagulants, and presence of diverticula on colonoscopy were not significant predictors of a FP-FIT result.

### Multiple Linear Regression Identifies Predictors of Adenomatous Polyp Count

Increasing age (*β* = 0.07, *p* = 0.000) and BMI (*β* = 0.04, *p* = 0.010) were associated with an increase in number of adenomatous polyps as demonstrated in Fig. 1. Female sex (*β* = − 0.66, *p* = 0.004) and the presence of hemorrhoids (*β* = − 0.52, *p* = 0.019) were associated with a decrease in the number of polyps compared to male sex and the absence of hemorrhoids.

### Predicting FP-FIT Using Machine Learning

**Fig. 2:**
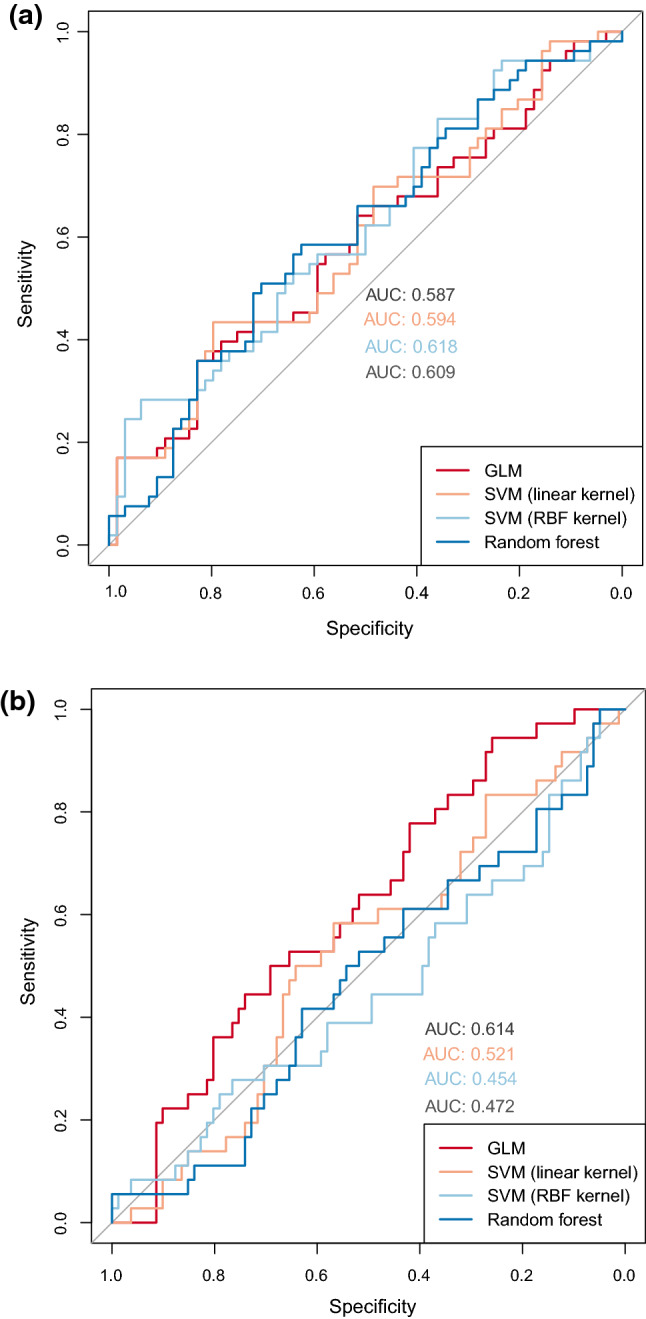
**a** Receiver operator characteristic curves for machine learning models trained to predict an FP-FIT result. **b** Receiver operator characteristic curves for machine learning models trained to predict the presence of advanced adenomas

Of the four supervised learning models trained to predict FP-FIT, the SVM with RBF kernel performed best with an AUROC of 0.618, as seen in Fig. 2a. At the optimal cut point determined using Youden’s index, FP-FIT was correctly identified 15 of 53 times, while TP-FIT was correctly identified 60 of 64 times. This yielded an accuracy of 64.1%, sensitivity of 28.3%, specificity of 93.8%, PPV of 79.0%, and NPV of 61.2%. At the optimal cut point determined using the point closest to (0,1), FP-FIT was correctly identified 28 of 53 times, while TP-FIT was correctly identified 41 of 64 times. This yielded an accuracy of 59.0%, sensitivity of 52.8%, specificity of 64.1%, PPV of 54.9%, and NPV of 62.1%.

### Predicting the Presence of Advanced Adenomas Using Machine Learning

Of the four supervised learning models trained to predict the presence of advanced adenomas, the GLM performed best with an AUROC of 0.614 (Fig. 2b). At the optimal cut point determined using Youden’s index, the presence of an advance adenoma was correctly predicted 34 of 36 times, while the absence of an advance adenoma was correctly predicted 21 of 81 times. This yielded an accuracy of 47.0%, sensitivity of 94.4%, specificity of 25.9%, PPV of 36.2%, and NPV of 91.3%. At the optimal cut point determined using the point closest to (0,1), the presence of an advanced adenoma was correctly identified in 19 of 36 cases, while the absence of advanced adenoma was correctly identified in 53 of 81 cases, yielding an accuracy of 61.5%, sensitivity of 52.8%, specificity of 65.4%, PPV of 40.4%, and NPV of 75.7%.

## Discussion

In this study, we examined factors associated with FP-FIT results and conversely, factors associated with the presence of adenomatous polyps. Our cohort included average-risk LADHS patients who had a positive result with the standard OC-Auto-FIT kit using a hemoglobin cutoff of 20 ug/g. The study identified 596 FIT-positive participants, 45% of whom were found to have a FP-FIT after undergoing colonoscopy. Given the large healthcare burden caused by FP-FIT results and subsequent colonoscopies, it is crucial to identify patients who may or may not have high diagnostic yield for CRC or advanced adenomas on colonoscopy.

Across the literature, there does not appear to be any consensus as to which factors are predictive of FP-FIT results. In studies from Germany and the Netherlands, male sex, older age, and greater BMI were significant predictors of a FP-FIT [9]. In contrast, studies from Barcelona, Australia, and Italy showed that older age and male sex were associated with increased odds of advanced neoplasia, thus a TP-FIT [15, 19, 21]. A large meta-analysis that included both Asian and European populations found female sex and NSAIDs to be significant predictors of FP-FIT [9]. In addition, hemorrhoids were found to increase odds of a FP-FIT in a large Korean cohort [13], though hemorrhoids were not found to be a significant predictor in studies from the Netherlands and Taiwan [5, 33, 34].

Within our largely Hispanic LADHS cohort, we found that female sex, younger age, lower BMI, and presence of hemorrhoids on colonoscopy significantly increased the odds of an FP-FIT result. Similarly, female sex and presence of hemorrhoids were associated with a fewer number of adenomatous polyps. These results are consistent with established data that male gender, older age, and higher BMI are known predictors for gastrointestinal malignancies [35–37]. Furthermore, the presence of hemorrhoids is a commonly suspected cause of FP-FIT as it is known to cause rectal bleeding. Finally, our findings that anticoagulants, antiplatelet agents, and NSAIDs do not affect FP-FIT results are in line with data from other studies [7–10].

We trained four MLMs to predict FP-FIT and four other MLMs to predict the presence of advanced adenomas. The SVM with RBF kernel demonstrated the best performance predicting an FP-FIT result, while the GLM demonstrated the best performance predicting the presence of advanced adenomas with AUROCs of 0.618 and 0.614 respectively. Given the low AUROCs, the MLMs do not perform well enough to be clinically valuable; however, retraining them using a larger dataset and a different set of features may improve their performance.

A primary strength of this study was the consistency of FIT testing, as all participants received the same OC-Auto-FIT test with a standard hemoglobin cutoff at 20 ug/g. Additionally, the multiple predictors individually studied in separate studies previously were evaluated collectively within this study. These factors included not only demographic data, but also clinical history, presence of diverticula or hemorrhoids on colonoscopy, as well as medication history with NSAIDs, antiplatelet use, or anticoagulation use. Finally, this study is one of the largest studies of FP-FIT involving a predominantly Hispanic, safety net population.

Despite the substantial strengths of this study, several limitations should be acknowledged. First, the study did not include patients with positive FIT results who did not attend their colonoscopy appointment or were lost to follow-up. Because these individuals did not have a diagnostic result after positive FIT, this could potentially affect selection bias. Second, the presence of hemorrhoids on colonoscopy predicted a FP-FIT. Notably, however, this information may not be known at time of FIT invitation, making it difficult for clinicians to predict FP-FIT. Third, we did not distinguish between initial FIT and repeated FIT testing, which can improve adenoma detection. Subgroup analysis of repeated applications of FIT beyond 1-time FIT may improve sensitivity of our FP-FIT MLM. Finally, the study did not examine the statistical impact of adenomas that may have been missed during colonoscopy.

Based on the findings in this study, clinicians can implement a risk-based screening strategy to determine which patients may have a low or high yield diagnostic colonoscopy following a positive FIT result. Pending further validation, these data may be useful in determining the most appropriate CRC screening modality in patients who are at increased a prior risk of a FP-FIT and when interpreting a positive FIT result. Particularly with the new US Preventive Services Task Force (USPSTF) proposal to initiate screening at age 45, gastroenterologists have an even greater responsibility to stratify which patients should be scheduled for colonoscopy and which patients can get FIT testing [38]. Overall, the addition of personalized, risk-based screening strategies could increase the accuracy and diagnostic yield of FIT screening, reducing the number of unnecessary colonoscopies and healthcare burdens.

## Abbreviations

BMI: Body mass index
GI: Gastrointestinal
NSAIDs: Nonsteroidal anti-inflammatory drugs
IQR: Interquartile range
